# The Capacity of Drug-Metabolising Enzymes in Modulating the Therapeutic Efficacy of Drugs to Treat Rhabdomyosarcoma

**DOI:** 10.3390/cancers16051012

**Published:** 2024-02-29

**Authors:** Enric Arasanz Picher, Muhammad Wahajuddin, Stefan Barth, Julia Chisholm, Janet Shipley, Klaus Pors

**Affiliations:** 1 Institute of Cancer Therapeutics, Faculty of Life Sciences, University of Bradford, Bradford BD7 1DP, UK; earasanz@bradford.ac.uk (E.A.P.); m.wahajuddin@bradford.ac.uk (M.W.); 2 Medical Biotechnology and Immunotherapy Research Unit, Institute of Infectious Disease and Molecular Medicine, Faculty of Health Sciences, University of Cape Town, Cape Town 7700, South Africa; stefan.barth@uct.ac.za; 3 Children and Young People’s Unit, Royal Marsden Hospital, Institute of Cancer Research, Sutton SM2 5PR, UK; julia.chisholm@rmh.nhs.uk; 4 Sarcoma Molecular Pathology Group, Division of Molecular Pathology, The Institute of Cancer Research, Sutton SM2 5NG, UK; janet.shipley@icr.ac.uk

**Keywords:** rhabdomyosarcoma, drug-metabolizing enzymes, cytochrome P450, aldehyde dehydrogenase, carboxyl esterase, cyclophosphamide, ifosfamide, irinotecan, ALDH, CYP450

## Abstract

**Simple Summary:**

Rhabdomyosarcoma is a rare type of childhood cancer. Current treatment options include surgery, radiotherapy, and chemotherapy. Survival chances for RMS patients have increased over the last few decades as a result of improved risk-based patient allocation procedures in therapeutic protocols. However, RMS patients bearing an advanced disease at diagnosis still fare badly, and mortality rates remain very high. The novel chemotherapeutic combinations tested in clinical trials for RMS have failed to improve outcomes in this subset of patients. The impact of drug-metabolising enzymes (DMEs) on drug efficacy and their potential as targets for targeted therapy are sometimes overlooked. Here, we critically review the literature and what has been achieved in the treatment of RMS, with a focus on how DMEs modify patients’ responses to therapy, and discuss new ideas towards the development of targeted therapies.

**Abstract:**

Rhabdomyosarcoma (RMS) is a rare soft tissue sarcoma (STS) that predominantly affects children and teenagers. It is the most common STS in children (40%) and accounts for 5–8% of total childhood malignancies. Apart from surgery and radiotherapy in eligible patients, standard chemotherapy is the only therapeutic option clinically available for RMS patients. While survival rates for this childhood cancer have considerably improved over the last few decades for low-risk and intermediate-risk cases, the mortality rate remains exceptionally high in high-risk RMS patients with recurrent and/or metastatic disease. The intensification of chemotherapeutic protocols in advanced-stage RMS has historically induced aggravated toxicity with only very modest therapeutic gain. In this review, we critically analyse what has been achieved so far in RMS therapy and provide insight into how a diverse group of drug-metabolising enzymes (DMEs) possess the capacity to modify the clinical efficacy of chemotherapy. We provide suggestions for new therapeutic strategies that exploit the presence of DMEs for prodrug activation, targeted chemotherapy that does not rely on DMEs, and RMS-molecular-subtype-targeted therapies that have the potential to enter clinical evaluation.

## 1. Introduction

Rhabdomyosarcoma (RMS) is a rare, skeletal muscle-like malignancy that principally arises in children and adolescents, although it occasionally occurs in adults. Despite showing a low prevalence, it is the most frequent soft-tissue sarcoma (STS) in children (around 40%) and accounts for around 5–8% of all childhood cancers [1]. More than 50% of RMS cases appear during the first decade of life, representing 3.5% of cancers between 0 and 14 years of age and around 2% of total cancers in older children (15–19 years) [2].

Critical differences exist between different RMS subtypes, which were first identified at a histological level, and allowed for the original classification of RMS into different tumour subtypes [3]. The two most common histological RMS subtypes are known as embryonal rhabdomyosarcoma (ERMS) and alveolar rhabdomyosarcoma (ARMS), which can be grouped into further distinctive genetic and molecular features. ERMS is the most common RMS subtype, predominantly occurring in the head, neck, and genitourinary regions [4]. It is genetically variable, harbouring multiple alterations in the DNA, but generally shows the best prognosis among all the subgroups. ERMS tumours tend to appear in younger children (<5 years old), but studies [5] have shown a second rise in ERMS cases in teenagers (12–17 years old), especially in males, indicating that ERMS might follow a bimodal epidemiological distribution [6].

ARMS is the second most prevalent RMS type. Its name is given owing to its alveolus-like morphology, containing interseptal structures between cells and resembling lung parenchyma. The age of onset at diagnosis is slightly higher than in ERMS, arising in older children and adolescents [7] and with the extremities and trunk as the most habitual primary sites [8]. These tumours possess a lower mutational rate, although a translocation is found in 80% of ARMS tumours, which results in PAX3/7-FOXO1 fusion protein formation. The end product of this novel genetic segment is thought to act as a chimeric oncogene, fostering tumorigenesis and increasing tumour malignancy and metastatic potential [7]. Consequently, the outcome in ARMS tends to be poorer than in ERMS [9,10].

In the 20% of ARMS cases where the translocation is absent, the resulting tumours appear to resemble, both molecularly and clinically, ERMS rather than ARMS disease [11]. A refinement of classifying RMS disease has recently appeared, which is replacing the classic histological sorting system (embryonal/alveolar), consisting of fusion-positive RMS (FPRMS) and fusion-negative RMS (FNRMS) systems. This new categorisation model is widely used in current research and more accurately illustrates tumour differences with respect to biological and medical parameters. FPRMS patients have, in 75% of cases, a characteristic balanced translocation, t(2;13)(q35;q14), rearranging the PAX3 (intron 7) and FOXO1 (intron 1) genes and creating the new fusion protein PAX3-FOXO1. In a minority of patients, these translocations happen between chromosome 1 (instead of chromosome 2) and the FOXO1 gene, which encodes the PAX7-FOXO1 fusion protein [12].

Pleomorphic rhabdomyosarcoma (PRMS) is an infrequent variant predominantly affecting adults [13]. It is usually treated according to adult sarcoma protocols and shows a poorer prognosis compared with the two previous subtypes. Its higher aggressiveness stems from its characteristic chemoresistance, which makes outcomes for patients largely dependent on surgery [14].

Clinical management in childhood cancer, including RMS with good prognosis, has experienced a dramatic improvement over the last decades. The introduction of chemotherapy increased initial 20–30% survival rates in the 1960s to as high as 62% in the 1970s, and the further improvement of therapies has improved survival chances up to >80% [15]. However, survival in high-risk patients (including with metastatic and relapsed RMS) remains very poor (<30%). Additionally, therapy intensification studies have also failed as no/little therapeutic index has been gained with increased side effects. The fact that there have only been modest improvements in outcomes for high-risk disease since the 1970s means that the main challenge remains the development of new strategies for the treatment of advanced-stage RMS.

Here, focusing on systemic therapy, we have briefly reviewed the evolution of RMS treatment, highlighting the key findings and advances in the clinical management of this disease and considering the impact of the latest clinical trials. In addition, we enumerate pharmacokinetic-related aspects that have an impact on drug efficacy in RMS patients, with special attention to drug-metabolising enzymes (DMEs), which might alter the effectiveness of clinically used drugs via the modulation of their structure to either reduce or potentiate the potency of the metabolites. Finally, we propose new insights that could pave the way towards better therapies and success in RMS treatment.

## 2. The Evolution of the Treatment for RMS and Recent Clinical Trials

### 2.1. Brief History of RMS Treatment

RMS follows the classical TNM staging system, which, alongside tumour size and anatomical site (favourable or unfavourable), defines the disease stage at diagnosis. After the initial surgical procedures are carried out, patients are assigned to a clinical group (IRS group) depending on the extent of tumour resection (I, complete resection; II, microscopic residual disease; III, macroscopic clinical disease; IV, metastatic disease). Treatment decision considerations include multiple variables, including stage, IRS group, histology or PAX-FOXO1 fusion status, and age, which are combined to define specific risk groups. Adolescent and young adult patients with RMS often have poorer outcomes than children. Accordingly, 10 years of age is used as a cut-off separating patients with a favourable prognosis (age < 10 years) from those with a worse predicted outcome (>10 years).

The current guidelines for RMS treatment involve the use of a multimodal, multiagent treatment, including surgery, radiation, and chemotherapy. Systemic chemotherapy based on a DNA-alkylating-based backbone approved in the 1970s has helped to eliminate possible dissemination and prevent tumour relapse. In Europe, IVA (ifosfamide + vincristine + actinomycin D) has been approved for clinical use for RMS treatment, while in North America, ifosfamide is replaced by cyclophosphamide (VAC). The omission of an alkylating agent in low-risk patients and the inclusion of doxorubicin in the treatment of high-risk patients has been applied in clinical practice. Moreover, irinotecan is used by the Children’s Oncology Group for intermediate-risk patients and is used as the standard of care in Europe in combination to treat relapsed patients. These drug combinations are the therapies that are taken as a reference for all RMS patients, although the number of drugs administered, cycles, and dosage will depend on the risk group of the patients, as well as differences between the major treating cooperative groups (European Paediatric Soft Tissue Sarcoma Study Group; Children’s Oncology Group; CWS Group).

**Figure 1:**
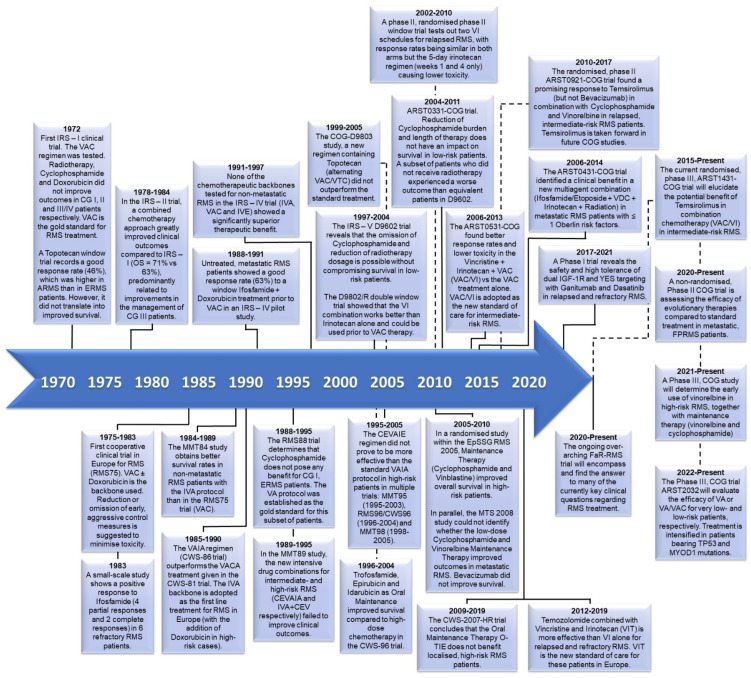
Key clinical trials that have shaped the evolution of RMS treatment over five decades since the 1970s. Timeline depicting key findings and significant clinical research in RMS over the last 5 decades. Studies are divided by geographical location: USA (top) or Europe (bottom). CEV—carboplatin, epirubicin, vincristine; CEVAIA—CEV + actinomycin D, ifosfamide, doxorubicin; CEVAIE—CEV + actinomycin D, ifosfamide, etoposide; IVE—ifosfamide, vincristine, etoposide; O-TIE—oral trofosfamide, idarubucin, epirubicin; VACA—VAC + doxorucibin; VAIA—IVA + doxorubicin; VI; VDC—vincristine, doxorubicin, cyclophosphamide; VTC—vincristine, topotecan, cyclophosphamide.

In 1972, the first Intergroup Rhabdomyosarcoma Study (IRS) opened, kicking off a series of cooperative group studies to improve and refine RMS treatment and cure rates in the US and, later, in Europe. Over the last couple of decades, advances have been made in terms of survival rates and side effects in low- and intermediate-risk RMS patients by reducing or omitting the alkylating agent’s cumulative dose and paying attention to local control measures (particularly radiotherapy). This has been achieved without compromising the therapeutic efficacy and has improved the risk stratification of patients. Together this led to the design of better patient-tailored treatment regimens [16]. However, the different therapeutic alternatives suggested for high-risk patients (including the intensification of existing treatments, the evaluation of new treatment schemes and drug combinations, and different maintenance therapy options) have resulted in modest outcomes. Figure 1 outlines a timeline for the most relevant clinical trials carried out, both currently and in the past, on RMS patients. For in-depth background knowledge, we refer readers to an excellent review by Arndt et al. [16].

### 2.2. Introduction of Other Marketed Drugs into Clinical Trial Combinations

Other drugs approved for clinical use have entered clinical trials to evaluate their efficacy in RMS patients, encompassing metastatic, recurrent, and relapsed disease. Compounds like the topoisomerase I inhibitor irinotecan, the alkylating agent temozolomide, the mTOR inhibitor temsirolimus, and monoclonal antibodies like cixutumumab (anti-IGF-1R) and bevacizumab (anti-VEGF) have been investigational drugs in these studies.

The pharmacological activation of irinotecan (or CPT-11) involves the participation of carboxylesterases (CE) to generate the active metabolite SN-38, a promising prodrug for the treatment of STS [17]. Earlier, both irinotecan and temozolomide proved to be effective in Ewing sarcoma clinical trials [18,19,20,21], and this encouraged the evaluation of these compounds in RMS. A phase II study from the STSC (2002–2004) revealed an interesting response rate of refractory and recurrent STS patients, including RMS, to irinotecan [22]. The ERMS subtype responded substantially better (1/4 partial responses and 3/4 minor responses) than ARMS (1/8 partial responses, 1/8 no response, 6/8 progressive disease). In a collaborative study between the French Society of Paediatric Oncology and the UK’s Children’s Cancer Study Group, irinotecan showed an interesting (11.4%) response rate in refractory RMS patients and a good tolerance profile [23]. The role of irinotecan as a radiosensitising agent was investigated in a phase II study including intermediate- and high-risk RMS patients [24]. Newly diagnosed patients were treated with carboplatin and irinotecan concurrently with radiotherapy prior to standard treatment, demonstrating promising efficacy and tolerance. Based on a reduction in side effects without compromising efficacy in the relapse setting [25], a 5-day schedule of irinotecan was included in the ARST0531-COG study (2006–2013), where VAC with a vincristine + irinotecan (VAC/VI) regimen showed the same 4-year FFS (62%) as the conventional VAC treatment in patients with intermediate-risk disease [26,27].

So far, temozolomide has been evaluated in RMS in combination with protocols containing vincristine and irinotecan. A retrospective, multicentre analysis of relapsed RMS patients detected a stable disease in 4/15 patients (26.7%) after treatment with vincristine, irinotecan (VI), and temozolomide, demonstrating this regimen as an option for salvage therapy [28]. A key phase II trial carried out by the EpSSG (VIT-0910, 2012–2019) compared the response to VI or VI + temozolomide (VIT) in children and adults with relapsed or refractory RMS. Response rates were 31% vs. 44%, overall survival was significantly higher in the VIT group compared with VI alone, and toxicity was manageable. Thus, this VIT arm is considered the new standard of care in Europe for this subset of patients [29]. However, neither temozolomide nor cixutumumab alone with the ARST0431 regimen (ARST08P1) showed additional efficacy compared with dose-compressed, intensive chemotherapy alone [30,31].

Temsirolimus inhibits the cell cycle regulator mTOR protein via interaction with the FKBP-12 protein, resulting in G1 phase cell arrest. This drug alone showed very limited efficacy in relapsed/refractory RMS patients (1 out of 16 patients) [32]. Nevertheless, in a posterior COG randomised, phase II trial (ARST0921), temsirolimus combined with vinorelbine and cyclophosphamide in intermediate-risk, first-relapsed RMS patients showed a 47% response rate and a low percentage of progressive disease at 6 weeks (11%). When temsirolimus was substituted by bevacizumab, response rates dropped (28%) while progressive disease increased (28%) [33]. Currently, the ARST1431-COG study is evaluating the potential therapeutic benefits of temsirolimus when combined with VAC/VI chemotherapy in intermediate-risk RMS patients (NCT02567435) after it proved to be safely administered to this group of patients [34].

The anti-IGF-1R monoclonal antibody Ganitumab showed good tolerance but limited therapeutic effect in a phase II, STS clinical trial including RMS patients [35]. Helman et al. characterized a resistance mechanism to IGF-1R inhibition with Ganitumab that consisted of the activation of the YES/SFK kinase in both in vitro and in vivo RMS models [36]. A recent phase I trial proved that the co-targeting of both pathways with Ganitumab and Dasatinib (multi-kinase inhibitor targeting YES/SFK) is safe and well tolerated by refractory/relapsed RMS patients (NCT03041701) [37]. The clinical benefit of this or similar dual treatments may be assessed in future trials, although the discontinuation of Ganitumab poses a challenge.

Of note, the active FaR-RMS clinical trial by the EpSSG (NCT04625907) is currently in the recruitment phase and is expected to address a wide range of clinical questions in both childhood and adult RMS involving radiotherapeutic protocols, changes in chemotherapy dosage, the role of prolonged maintenance therapy, the new fusion protein-based risk stratification method, and testing new chemotherapeutic regimens depending on emerging data. Other clinical trials are currently active or accepting patients. These include the phase III ARST2032 trial (NCT05304585), investigating the survival of low-risk and very low-risk RMS patients when treated with VA/VAC or VA alone, respectively. Additionally, the benefits of treatment intensification in MYOD1- or TP53-mutated tumours will be tested. Another non-randomised COG study (NCT04388839) will focus on the efficacy of an evolutionary therapy for RMS, comparing metastatic FPRMS patients treated with either a conventional VAC treatment, a VAC treatment plus maintenance therapy (vinorelbine and cyclophosphamide), or a VAC adaptive therapy (based on responses). In fact, a meta-analysis encompassing five phase II trials revealed better responses to vinorelbine in ARMS patients than in ERMS patients [38]. Finally, a COG phase III study is currently assessing the early use of vinorelbine in high-risk RMS patients (NCT04994132). Patients will be allocated to a VAC + maintenance therapy (vinorelbine and cyclophosphamide) arm or to a VINO AC + maintenance therapy arm (where vincristine is replaced by vinorelbine, and maintenance therapy remains the same).

## 3. Contributing Factors That Reduce Drug Efficacy

Despite the arrival of precision medicine, chemotherapy, as indicated above, still represents the mainstay for the standard treatment of RMS. Extensive clinical trials have provided insight into the best use of drug combinations in Europe and in the US [16], yet not much work has been devoted to analysing their limitations. It is well-established that tumours harbour intrinsic and/or acquired resistance mechanisms that modulate the efficacy of a drug. Cross-resistance often follows and negatively impacts drug treatment combinations. In the following sections, we focus on pharmacokinetic-related aspects and, in particular, drug-metabolising enzymes (DMEs) that alter the drug structure to either reduce or potentiate the potency of metabolites.

### 3.1. Pharmacodynamics- and Pharmacokinetics-Related Critical Insight Observations

Developmental changes in physiologic processes occur during adolescence and lead to highly variable alterations in drug disposition in this age group. Factors that can influence treatment outcomes include absorption, distribution, metabolism, and elimination (ADME) [39]. Small-molecule drugs differ in their structural makeup, which affects their ADME properties; pharmacokinetics; and, ultimately, drug efficacy. Ifosfamide and cyclophosphamide are oxazaphosphorine-based prodrugs that need to be activated metabolically to exert their pharmacodynamic effects. Their therapeutic use is hampered by the dose-limiting toxicity of haemorrhagic cystitis; however, this can be reduced via co-administration with mesna, which ameliorates both hyperhydration and the metabolic profile of urotoxic by-products from these prodrugs via reaction and detoxification with, e.g., acrolein [40]. Ifosfamide shows dose-independent pharmacokinetics with faster absorption upon administration, reaching the maximum concentration within one hour. Furthermore, the volume of distribution is enhanced in obese and elderly patients, which may be due to increased body fat. The terminal half-life after oral and intravenous administration has been estimated to be between 4 and 7 h [41]. Similarly, cyclophosphamide has been shown to have an elimination half-life that ranges between 5 and 9 h in children and young adults, shorter than in adults which might be related to enhanced metabolic activity [42].

The renal clearance of ifosfamide and cyclophosphamide has been found to be reliant on urine flow rate, with increased hydration correlating with the enhanced renal clearance of both these therapeutic agents. Currently, there is no clear-cut evidence that ifosfamide has any therapeutic merit over using cyclophosphamide in this combination. Indeed, there is not much information available on the clinical significance of drug-to-drug interactions involving these agents. Potentially, the co-administered agents may influence the metabolism of these drugs [41,42].

The clinical pharmacokinetics of vincristine has been estimated to be highly variable and unpredictable, with therapeutic outcomes largely influenced by co-administered medication [43,44]. Vincristine pharmacokinetics is not age-dependent in this paediatric population, with no pharmacokinetic rationale for dose reduction in adolescents [45]. Additionally, increased neurotoxicity manifesting as vinca-alkaloid-induced peripheral neuropathy, despite similar hematologic toxicity when comparing older and younger patients with RMS, makes it debatable as to what extent side effect toxicity plays a role in treatment outcomes observed [46,47]. Similarly, actinomycin D has also demonstrated highly variable pharmacokinetics that is still indecipherable even after taking into consideration body weight, which is among the major determinants in clearance [48].

A pharmacokinetic analysis of topotecan, an analogue of irinotecan, was focused on investigating the stability of the pharmacophore-located lactone in 162 children enrolled in six phase I/II clinical trials. Insights provided by these studies suggested body surface area (BSA) was related to topotecan clearance, potentially linking treatment outcomes to inter-individual variability. However, no differences in systemic clearance were noted between adolescents and younger children [49], perhaps suggesting that BSA is more important than any metabolic or hydrolytic degradation of the lactone ring. Similarly, a study of 98 children in the age range of 1–17 years showed no relationship between age and vincristine pharmacokinetics [45]. It should be noted that this study was focused on acute lymphoblastic leukaemia; hence, vincristine cancer cell uptake is superior compared with, e.g., solid RMS uptake, so the pharmacokinetics and drug efficacy are likely to differ significantly.

### 3.2. Drug-Metabolising Enzyme (DME) Expression and Activity

Any drug administered to the body inevitably undergoes a metabolic process that will change its chemical structure and determine its distribution efficacy, clearance, and safety. The enzymes capable of catalysing these chemical reactions are DMEs, which are extensively expressed in the liver and, to a lesser degree, the kidney and small intestine [50]. Active drugs can be inactivated via metabolism, whereas prodrugs are designed to be administered in an inactive, innocuous form so that their cytotoxic potential is unleashed after metabolism. DME activity is often linked to transporter proteins that affect therapeutic efficacy. The importance of drug transporters cannot be ignored, and rather than discussing this in great detail here, we refer readers to an in-depth review of drug transporters in paediatric tumours [51].

The expression of DMEs undergoes critical changes during childhood and adolescence [52]. Accordingly, the impact on the activity of these enzymes is likely to affect the clinical response of infants and youngsters to drugs at multiple levels. Reportedly, DMEs can be broadly grouped into three different developmental patterns: (i) the ‘prenatal pattern’ makes reference to DMEs whose expression and activity are abundant during foetal phases, but their activity is intrinsically reduced after birth, indicating they play key roles during gestation for essential foetal development; (ii) the ‘constant pattern’ includes DMEs that show a stable, basal expression and activity at both prenatal and postnatal stages, regardless of the age of the individual; (iii) the ‘postnatal pattern’ covers a group of DMEs that are silenced and inactive during pregnancy, but their activity increases over the very first years of life and remains fully active during adulthood. Monooxygenases like CYP3A4, a key enzyme for the bioactivation of ifosfamide and cyclophosphamide, belong to this latter expression pattern. This information should be considered when treating RMS patients with alkylating agents, including oxaphosphorines, as their efficacy could be severely reduced, especially in very young individuals (<2 years old), when CYP3A4 expression is still immature. A peak of activity in the latter group is registered in toddlers (2–4 years old), probably because of a higher liver mass/total body mass ratio [53] (and the resultant overall increased metabolism), as no evidence of incremented enzymatic activity has been found. These patterns confirm in vivo data indicating that drug clearance is lower in neonates (and, thus, has a higher risk of toxicities), experiencing a peak around 2–10 years of age and reaching adult levels by adolescence. Indeed, some DMEs carry out physiological functions in metabolism, having endogenous compounds as substrates. Eventually, this ontogenic process culminates in late adolescence or adulthood, when stability in DME activity from all three groups is achieved [52]. Improvement in RMS treatment could be made through studies meant to understand DME expression in paediatric patients. Ideally, the analysis of DME expression levels in normal tissue—such as the liver and kidneys versus in RMS prior to the selection of a treatment protocol—could help to select patients that are affected positively or negatively by, e.g., high or low levels of prodrug-activating enzymes from classes of enzymes such as cytochrome P450 and carboxylesterases.

The biggest changes in DME activity take place during the neonatal and childhood periods when the metabolic system is maturing. This prompts serious challenges in the treatment of childhood cancer patients, as the overall response to drugs by the patient can be rather uncertain. An illustrative example is the use of antibiotic chloramphenicol in neonates with sepsis, which was shown to cause the deadly grey baby syndrome back in the 1950s. Specifically, this baby syndrome was a result of toxic drug accumulation in the body due to a lack of functional activity in UDP-Glucuronosyltransferase-2B7 (UGT2B7), a phase II DME whose enzymatic activity has been linked to chloramphenicol metabolism [54]. Similarly, ifosfamide-induced toxicity is exacerbated because of accentuated CYP3A activity, leading to the formation of toxic metabolites in normal tissues. Conversely, neonates are less susceptible to developing liver toxicity to paracetamol because of limited CYP2E1 activity. Importantly, DME activity does appear to be similar in teenagers and adults, suggesting that dosing recommendations for adults may be applicable to adolescents. For instance, drug glucuronidation patterns tend to be clearly differentiated between neonates and young children compared with teenagers and adults, such as in the case of acetaminophen [55]. Nevertheless, in younger patients, age-adjusted dosing guidelines are necessary in accordance with DME ontogeny (developmental patterns). Some drugs could even be ruled out for application in children, while some other agents that are toxic in adults might be suitable for use in paediatrics.

As discussed above, irinotecan is a comparatively new drug used as a part of various treatment regimens. In COG, it is used up front, while in the EpSSG, it is used to treat relapsed tumours. In the FaR-RMS study, irinotecan is used in combination with IVA, which is currently being investigated in phase Ib. It will also be evaluated in upfront randomisations in high-risk and very high-risk patients once the recommended phase II dose (in combination with IVA) is known.

Irinotecan is a prodrug that is reported to have complex metabolism involving several phase 1 and 2 metabolic enzymes leading to variable pharmacokinetics [56]. Irinotecan prodrug activation is key to efficacy, as it results in the formation of a log-scale-fold more active metabolite, SN-38. Irinotecan has been recommended for dose adjustments while co-administered with CYP3A4 inducers or inhibitors [57]. Clinical use has been approved in a wide spectrum of adult and childhood cancers despite its activation not being tumour-selective. However, a recent in vivo study showed that irinotecan is potentiated in a model that used neural stem cells carrying recombinant carboxylesterases, suggesting the increased bioactivation of target enzymes in tumour tissue [58]. Nevertheless, the delivery of the enzyme of interest to the tumour tissue remains a challenge in cancers that do not express a targetable DME.

#### 3.2.1. CYP-Mediated Drug Metabolism in RMS Patients

Cytochrome P450 (CYP) enzymes belong to a very large and heterogeneous family of heme-containing proteins that constitute one of the most important groups of metabolic enzymes in humans. This family of monooxygenases, which includes 57 human genes and 59 pseudogenes [59], fulfils many metabolic tasks, which essentially can be broken down into three major functions: (i) the metabolism of endogenous substrates, (ii) drug and xenobiotic metabolism, and (iii) the bioactivation of environmental procarcinogens [60]. They are also known as moonlighting proteins, meaning that they accomplish multiple functions through multiple catalytic sites, which can be unrelated and can happen in different tissues. The substantial homology existing between distinct CYP enzymes helps to explain the frequent substrate overlap among them. Thereby, one drug, chemical, or molecule can be simultaneously processed by more than one of the CYP isoforms, and a lone isoform can metabolise multiple substrates [61]. CYP1-4 families are responsible for xenobiotic metabolism and drug detoxification, as well as other endogenous molecules [60,62]. They are involved in approximately 80% of all phase I biotransformation reactions [63] and, hence, are critical components to consider in the drug development process. The CYP1-4 enzymes have been shown to both metabolically activate or inactivate chemotherapeutic agents [64]; the former is important for prodrug bioactivation and will be discussed further below. Thus, CYP activity greatly influences the success of therapeutic treatments in cancer and the degree of toxicity. Adverse drug reactions can appear sometimes when CYP active drug products skip phase II biotransformation conjugating reactions, where the active metabolite is transformed into less active ones [60].

The majority of CYP enzymatic activity takes place in the liver, but extrahepatic CYP isozyme expression also exists in a tissue-specific way [64]. Clinically, side effects are linked to the off-target bioactivation of prodrugs in non-malignant tissues. Likewise, targeted approaches can also be generated when prodrugs are designed to be specifically activated in the CYP-expressing tissue of interest, increasing in situ drug bioavailability and diminishing adverse effects [65]. The same principle can be employed when CYP expression arises in malignant tissues, where their presence offers an opportunity for therapeutic intervention [66,67]. Likewise, localised CYP activity in tumour cells can have a deleterious pharmacologic effect when active drugs are inactivated by these enzymes, leading to drug resistance; this, in turn, contributes to reduced drug doses in the tumour tissue and longer-term cancer recurrence. Furthermore, the levels of CYP isoform expression, genetic polymorphic variants, and metabolic rates contribute to both activation and inactivation mechanisms, influencing responses to therapy [68].

The impact of CYPs on the metabolism of first-choice anticancer agents has been reviewed for many tumour types [69,70] but not for rare diseases such as RMS. With respect to the compounds utilised in chemotherapeutic regimens for RMS treatment (VAC or IVA), the therapeutic success of both ifosfamide and cyclophosphamide depends largely on the bioactivation of these prodrugs caused by hepatic CYPs. Ifosfamide’s alkylating capacity is triggered by CYP2B6 and 3A4 metabolism with other minor contributors [71,72]. As far as cyclophosphamide is concerned, CYP2B6 and 3A4/5 are the main activators with the complementary participation of CYP2C subfamily isoforms [73,74,75]. A severe side effect of oxazaphosphorine-based prodrugs is hyponatremia. Given the high CYP2B6 expression in the kidneys and the cyclophosphamide-induced expression of aquaporins, not only is the expression of this isoform pharmacologically relevant but so are its tissue distribution and transcriptional effects [76].

As exemplified by the DNA-targeting prodrugs ifosfamide, cyclophosphamide, and irinotecan, the presence of DMEs can be exploited for therapeutic gain. Whereas ifosfamide and cyclophosphamide rely on CYP isoforms for bioactivation, irinotecan (CPT-11) is activated by carboxylesterases [77,78,79] to the active metabolite SN-38, which intercalates DNA, followed by subsequent topoisomerase I inhibition. Whereas CYP3A4/5 contribute to inactivation and the increased excretion rate of irinotecan, UGT1A1 forms glucuronidates, inactive products from SN-38 that are meant for excretion [57].

The elimination of vincristine and the circumvention of related side effects are thought largely to rely on CYP3A5-induced biotransformation, although CYP3A4 is also known to cooperate, albeit to a lesser extent [80]. Resistance to vinca alkaloids like vincristine has been linked to CYP3A4-mediated metabolism and the detoxification of these drugs [81,82].

Despite the importance of the CYP-activated oxazaphosphorine-based prodrugs ifosfamide and cyclophosphamide in RMS treatment, very few studies to date have attempted to characterise CYP expression in RMS models. This paucity of data emerges from its rather low epidemiological impact and shortage of clinical samples. Given the frequent clinical use of these CYP-activated prodrugs, it would be of potential clinical importance to understand whether CYP expression in RMS tissue can be used to enhance the activity of the oxazaphosphorine-based prodrugs, especially if these drugs could be delivered at higher concentrations via, for example, nano-formulated technologies [83,84].

A small-scale work by Molina-Ortiz et al. [85] is possibly the most relevant of the very few contributions in the area. The authors compared mRNA CYP450 expression patterns and protein levels between tumours and matched healthy tissues. The results suggested the unstable mRNA expression of CYP1A1 and CYP1A2 with negligible expression at the protein level. CYP1B1 and CYP2E1 showed steady mRNA amounts in both normal and tumour samples, but they did not correlate with protein levels in the latter. CYP3A4 and CYP3A5 transcripts and protein levels were higher in tumour tissue as compared with controls, but they were rather intermittent between patients. Only CYP2W1 displayed substantially increased mRNA (8/13) and protein (4/4) amounts in tumour samples specifically. mRNA levels were consistent with protein expression and strikingly, all the ERMS-derived tissues presented a high number of mRNA CYP2W1 transcripts. CYP2W1 expression is absent in normal tissue and elevated in several cancer types, including colorectal cancer [86,87,88], thereby providing a potential target for local drug activation in cancer tissues [88,89,90]. The Molina-Ortiz study only used 13 patient-derived RMS samples, and hence, a larger study is required before CYP2W1 can be validated and pursued as a target in RMS for therapeutic intervention.

Given the shortage of clinical tissue and experimental data, the examination of CYP expression in related malignancies (e.g., STS, childhood cancers) can be employed as a valuable method of extrapolation in RMS. Immunohistochemical analysis has revealed abundant CYP1A and CYP3A detection (70% and 78%, respectively) in a mixture of 37 adult STS samples [91]. Similarly, another publication that included young patients claimed that the CYP3A4 enzyme was present in most soft tissue Ewing sarcoma patients (81%), while CYP3A5 and CYP3A7 tumour expressions were fainter, in that order. Moreover, CYP3A4 acted as a metastatic marker [92]. In neuroblastoma, a classical paediatric disease, AhR-regulated CYP1A1 and CYP1B1 levels seem to increase upon exposure to inducer pollutants, and high expression correlates with better prognosis [93,94]. Even if CYP3A4 and CYP3A5 polymorphisms are supposed to influence survival in patients with neuroblastoma survival, larger studies are needed to validate it and to measure the actual expression levels [95]. Osteosarcoma is the most frequent bone tumour in children, and CYP1A1/2, CYP1B1, and CYP3A4/5 expressions have been commonly found in primary biopsies, the latter positively correlating with metastatic disease [96]. Later, CYP1A2 activity is reported to be important for lung metastasis, and CYP3A4/5 expression is low in biopsies but increased after treatment, especially in patients with favourable prognoses. In fact, CYP3A4 and CYP3A5 levels decreased and increased, respectively, in metastatic osteosarcoma samples in [97]. These differences might respond to the CYP regulatory activity of chemotherapeutic drugs, as it was shown in vitro in the same study. Such inconsistencies reflect the limitations of the former osteosarcoma study, the small sample being the most evident. For acute lymphoblastic leukaemia (ALL), the most common malignancy in childhood, CYP1A1 and CYP2D6 genotypes determine ALL risk [98].

#### 3.2.2. Aldehyde Dehydrogenases: Do They Play a Role in RMS Responses to Treatment?

**Figure 2:**
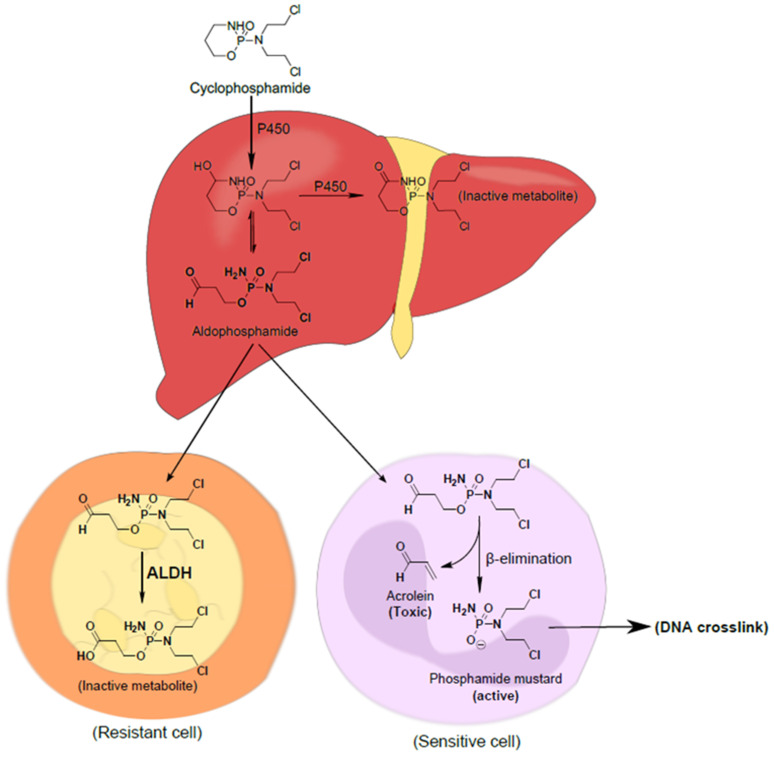
Cytochrome P450 (CYP) activation of cyclophosphamide. Initial hydroxylation of cyclophosphamide in the liver by CYP isoforms leads to the generation of aldophosphamide, an intermediate which is a substrate for ALDH metabolism. If aldophosphamide enters circulation, it is very likely to be detoxified in ALDH-expressing cells in both normal and cancerous tissue. (Reprinted with permission from Ibrahim et al. [108].)

The presence of DMEs such as CYPs and carboxylesterases is critical to prodrug activation and the effective treatment of RMS but also has implications for the reduced therapeutic index of cytotoxic agents such as vincristine and doxorubicin. Additionally, and perhaps historically less considered, there is the expression of another class of DMEs known as aldehyde dehydrogenases (ALDHs). The ALDH superfamily comprises 19 ALDH human subtypes, which are located in the cytoplasm, endoplasmic reticulum, mitochondria, and nucleus [99,100]. In the body, most ALDHs are widely distributed and found in the liver and kidney in high concentrations [101]. Apart from their functions as enzymes that detoxify endogenous and xenobiotic aldehydes, ALDHs also have specific biological roles in cells, such as in retinoic acid signalling, which is essential in embryogenesis and development [101]. The presence of ALDHs in tumours has been the subject of biomarker and drug development [102,103,104]. ALDH expression as biomarkers are linked to elevated expression profiles in cancer stem cell (CSC) populations [105,106,107,108] while specific isoform expression, despite normal tissue expression, is also considered and explored for therapeutic intervention [103]. Regarding RMS treatment, to what extent ALDH expression impacts the efficacy of the prodrugs cyclophosphamide and ifosfamide is poorly understood. Their prodrug activation involves the generation of aldophosphamide, an intermediate that is a substrate for ALDHs and which is likely to reduce the tumour concentration of DNA-damaging nitrogen mustard metabolites [103,108] (Figure 2). Not only is off-target detoxification in the liver an issue but ALDH-positive CSC subpopulations are likely to be highly resistant to these prodrugs and, hence, will survive treatment and contribute to tumour reoccurrence. The CYP-mediated activation of cyclophosphamide to phosphamide mustard (Figure 2) has been shown to represent a major metabolic route (70%), while 5% of cyclophosphamide is N-dechloroethylated by CYP3A4 into an inactive compound [109,110]. ALDH1 and ALDH3 have been identified with cellular resistance to cyclophosphamide, with both sub-classes contributing equally to the drug metabolism [111], although it has been shown that the catalytic rates of ALDH1A1 are superior to ALDH3A1 in reacting with aldophosphamide [112]. New efforts to improve therapeutic outcomes could also involve the inclusion of an ALDH inhibitor as a part of a combination treatment to sensitise cancer cells to standard-of-care drugs, including cyclophosphamide [113,114].

Although much work has been devoted to understanding RMS biology, more research is required to understand how the RMS microenvironment affects intracellular mechanisms, including DME expression, functional activity, and drug treatment. Consequently, deeper insight into the RMS microenvironment could not only more accurately reveal treatment limitations but also potentially provide opportunities to molecularly stratify patients for specific combination treatments. The RMS microenvironment features a hypoxic signature [115,116] that must be considered carefully when pursuing new knowledge to better understand RMS biology and/or treatment sensitivity/resistance mechanisms [117,118]. For example, emerging evidence indicates that CSCs might reside in the hypoxic niche, where they are believed to be more chemo- and radioprotected [119,120]. High ALDH expression in CSCs significantly reduces the drug effectiveness of both cyclophosphamide and ifosfamide, as mechanistically outlined in Figure 2. In other tumour types, it has been demonstrated that both chemo- and radiotherapy have the propensity to enrich the CSC subpopulation following treatment, which is likely to enhance drug resistance mechanisms and patient relapse [121]. To improve our understanding, we suggest a careful selection of preclinical models to support efforts by researchers with an interest in improving the knowledge of rare childhood cancer diseases such as RMS. In addition to long-established RMS cell line cultures, preclinical models include the use of recently derived models from patients grown directly in 2D and 3D cultures or models established in mice or zebra fish for direct in vivo use or subsequent growth in vitro. Furthermore, the addition of other cell types, such as cancer-associated fibroblasts (CAFs) and/or cells of the immune system, is becoming possible to include in modelling the tumour microenvironment (TME). Re-creating physiological conditions such as hypoxia through the use of hypoxic chambers or 3D spheroids of a large size that recapitulate hypoxia in solid tumours is also suitable for addressing specific questions.

## 4. Discussion and Future Directions

### 4.1. Pharmacokinetic Considerations and DME Ontogeny

As highlighted in this review, a patient’s unique paediatric physiology and pathology have implications for therapeutic outcomes. Dosage, pharmacokinetic sampling time points, and individual tolerance toxicity profiles make comparisons between young patients of different ages, and especially adults, difficult to ascertain. This encourages the use of a pragmatic approach in paediatric clinical trial design and endpoint determination and not merely following the tools and techniques used in clinical trials in adult populations.

Developmental biology poses challenges in the form of physiological changes in the short-term that are related to the age of a child and that show significant changes in pharmacokinetics, pharmacodynamics, tolerability, metabolism, immune response, and microenvironment at the site of action and at an organismic level. The criteria for choosing agents to investigate should take into account the crucial role of drug targets in paediatric cancer pathology and not just their expression in childhood cancer. Paediatric clinical trial designs should include a focus on cancer heterogeneity with the help of modern tools and diagnostics to stratify the childhood cancer subtypes together with the development of targeted chemotherapeutics.

Studies to elucidate the ontogeny and developmental patterns of different DMEs are predominantly performed in animal models. In vitro research is hampered by the scarcity of suitable biological tissue material available from children, as well as the restrictive regulatory hindrances for its collection. In vivo studies involve the administration of isotopically labelled, enzyme-specific probes to compare the abundance of metabolites in urine and blood so that the activity of individual enzymes in the patient can be monitored [122]. More studies like that of Hines and McCarver [123,124] are needed to shed light on DMEs; the investigators collected over 200 paediatric liver samples, which provided some much-needed evidence of phase I and II DME expression in children. A better understanding of the spatial–temporal maturational pattern of these enzymes throughout childhood and adolescence would provide the opportunity to better anticipate responses to drugs and develop individualised, more predictive models in the clinical setting, eventually optimising therapy in young patients. Additionally, other covariates might include a certain degree of variability, such as genetic variants (pharmacogenetics), comorbidities, growth and sex hormones, or effects from previous treatments [125].

### 4.2. The Impact of Drug Resistance Mechanisms on RMS

Since the 1970s, there have been many clinical studies aimed at understanding the best treatment combinations for combatting RMS. Advances and improvements in RMS treatment are principally attributed to (i) the discovery and implementation of more effective chemotherapeutic drug combinations; (ii) dose-tailored regimens to reduce therapeutic burden and life-threatening toxicities; and (iii) the optimisation of risk stratification schemes to better allocate patients into the appropriate treatment groups for chemotherapy, radiotherapy, and surgery. Despite clinical evaluations of new drugs to treat this subset of individuals, new treatments that outperform existing standard-of-care protocols have been limited to vinorelbine and continuous low-dose cyclophosphamide as a form of maintenance chemotherapy and the addition of temozolomide to vincristine and irinotecan at relapse [29,126].

**Figure 3:**
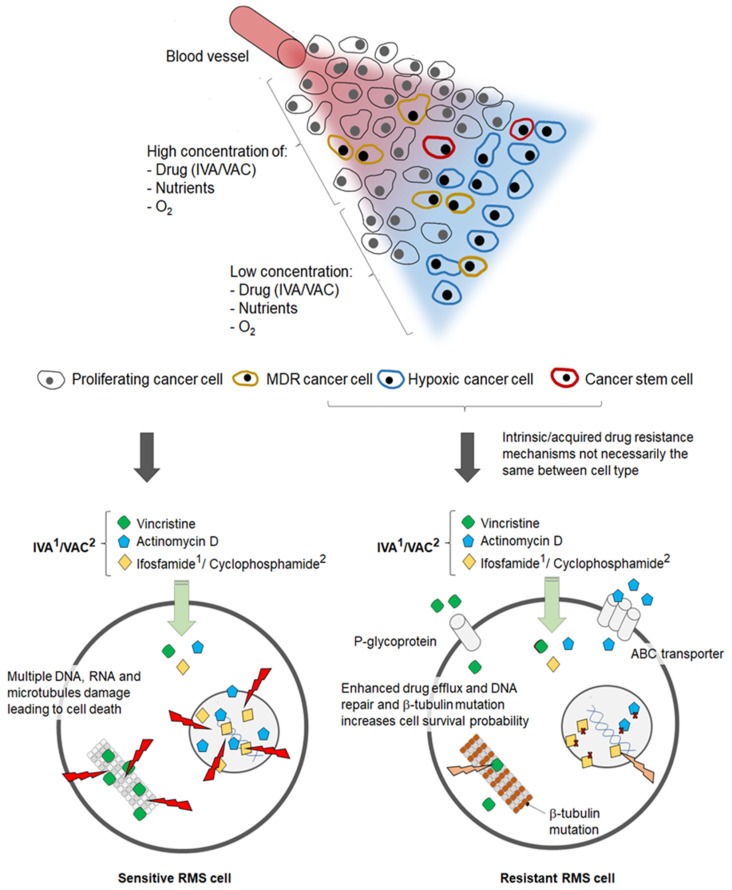
The tumour microenvironment and the presence of intrinsic and/or acquired resistance mechanisms impact treatment outcomes of chemotherapeutic drugs employed to treat rhabdomyosarcoma; the figure only outlines resistance to IVA and VAC treatment regimens.

The mechanism of action and drug resistance profiles of each chemotherapeutic drug used to treat RMS in the clinic have been demonstrated in many studies; however, less is understood about the resistance that might limit the effectiveness of combination regimens such as IVA and VAC; does the resistance profile change when provided as a low or high dose, or does the order of administration of the drugs matter? Critically, none of the multiple-drug treatment regimens that have been studied in the clinic (primarily IVA, VAC, VTC, VACA, VAIA, EVAIA, and CEVAIE) are tumour-selective. These are drug cocktails aimed at targeting dividing cells and, hence, are inherently associated with severe side effects. Patients with sensitive RMS disease can be treated successfully, whereas patients with resistant RMS tumours or cancers with subpopulations of resistant RMS cells are not exposed to high enough drug concentrations for complete tumour destruction. Figure 3 outlines multiple drug resistance mechanisms for IVA and VAC, as exemplars for RMS treatment regimens. Factors contributing to drug resistance include the presence of hypoxic cells distant from the blood vessels, a lack of efficacy in intrinsic or acquired multi-drug-resistant (MDR) cells, and the presence of CSCs that might have been enriched following the original cycles of chemotherapy.

For those patients who survive RMS disease, it would be insightful to measure DME expression before and after treatment (short- and long-term) to better understand the clinical impact of DME. This is especially important for patients who survive RMS disease and who might live with debilitating short- or long-term effects. These patients may harbour residual disease, leading to a risk of relapse due to acquired resistance or secondary tumours arising due to mutagenic and/or carcinogenic alterations induced by the DNA-targeted drugs employed.

Resistance to treatment is a major setback in RMS treatment and can lead to local or systemic relapse. Tumours are complex and consist of a heterogeneous mass of cells that dynamically adapts to therapeutic pressures upon treatment. Numerous are the reasons why some cells acquire resistance or survive frontline therapies, encompassing genetics; epigenetics; tumour cell localisation that affects the level of exposure to drugs; and pathway cross-talking within and between cells in the TME. Despite its involvement in drug resistance, the elevation in intratumoural DME expression offers an opportunity to be exploited for therapeutic gain, as discussed in recent reviews [58,70,102,104].

### 4.3. Exploitation of Genetic and Epigenetic Vulnerabilities for Therapeutic Intervention in RMS

Genetic changes in adult cancers are considered a reflection of chromosomal instability and regarded as a product of randomness that has been subjected to selective pressures. However, since the mutational rate is low in RMS (in FNRMS particularly) compared with other tumours, the non-genetic factors are expected to gain importance in the promotion of resistance [127]. A deeper understanding of the epigenetic landscape is needed for the identification of oncogenic drivers undisclosed by nucleic acid sequencing techniques. Eventually, only those cells that are resistant will persist and proliferate after treatment, resulting in incomplete tumour regression or minimal residual disease and recurrence, with the possibility of a more aggressive tumour phenotype emerging. Intermittent dosing approaches are used to try and combat tumour resistance and are based on the alternation of drug administration and withdrawal periods so that drug-sensitive tumour cells can repopulate the tumour [128]. However, it is arguable whether these methods will ever manage to kill resistant cells after the first drug treatment, and long periods of withdrawal may favour tumour progression. A second strategy that requires a great deal of preclinical and clinical testing is the design and use of combination therapies, targeting multiple vulnerabilities at a time. Resistant cells might be combated and eliminated via synthetic lethality approaches [129], although research in this area for RMS is sparse.

Shedding further light on the clear biological differences between RMS in adults versus children could lead to the therapeutic exploitation of the differences between histologic subtypes [46]. To address current drawbacks in treating patients in the high-risk groups, a focus should be shifted to moving newer RMS-targeted therapies closer to clinical evaluation, which have either benefitted from enhanced RMS disease knowledge or are designed to exploit RMS genetic vulnerabilities and opportunities existing in the TME. The role of patient-specific differences in these TMEs is reflected in a review describing recent trends in the immunotherapy of paediatric sarcomas [130]. As consistent checkpoint blockade therapies are currently missing, the identification of predictive biomarkers of response has been recognised as a prerequisite for future T cell-based immunotherapies. In addition to approaches aimed at regulating the activity of immune effector cells in the TME, these authors are also highlighting the use of neoantigen-displaying MHC receptors alongside differentially overexpressed differentiation antigens, including CD99, CD248, EGFR, GD2, HER2, and IGF1R, for selective target cell killing via, e.g., CAR-T cells or antibody–drug conjugates (ADCs), including recombinant immunotoxins. A recent surfaceome-profiling study based on the differential centrifugation enrichment of surface/membrane proteins and detection via LC-MS of six fusion-positive RMS cell lines, five fusion-negative RMS cell lines, and three RMS patient-derived xenografts (PDXs) indicated a range of potential cell surface targets for RMS immunotherapies, providing opportunities for new drug development strategies [131]. ADCs represent one of the fastest-growing drug delivery systems, a technology that involves a cancer-cell-targeted monoclonal antibody conjugated with a synthetic cytotoxic payload via a linker. In principle, an ADC reduces systemic exposure and provides site-specific delivery along with reduced toxicity. This approach could be applied to RMS and, e.g., include the specific delivery of a topoisomerase I inhibitor, as demonstrated for the two recent clinically approved ADCs Enhertu or Trodelvi, which would provide an attractive option for circumventing non-tumour prodrug activation via carboxylesterases [132]. Although more work is needed to understand how the disease-specific differential expression of such cell surface antigens can benefit RMS patients, they have a great potential to eventually unlock further immunotherapeutic strategies, whether alone or in combination with others [131,133].

Despite displaying clear distinctions in both genetic and molecular terms, FNRMS and FPRMS are still treated similarly. Investment and research to find new targeted therapies are needed so that, either alone or in combination with conventional therapies, a rational approach to treating high-risk RMS patients can be used to improve survival. Also, because RMS mostly affects children, all RMS survivors are long-term survivors; hence, the alleviation and mitigation of therapy-related, off-target, and long-term secondary effects to which children seem to be most vulnerable [15] is of utmost importance. It is expected that the gain of knowledge in the molecular biology, immune functionality, and pathophysiology of RMS will give rise to targeted, tumour-selective therapies that will overcome these issues. Novel RMS-selective treatments targeting genomic/epigenetic phenotypes and ARMS/ERMS/FNRMS/FPRMS molecular subtypes, e.g., distinctive fusion proteins such as PAX3-FOXO1 or MYOD1 mutant cells, could lead to less harmful and more effective treatment options for patients at high risk of succumbing to their disease.

## 5. Summary and Conclusions

The introduction of chemotherapy in the 1960s dramatically improved the prognosis of childhood RMS patients. Since then, the implementation and improvement of risk-stratified treatment allocations, as well as better dose-tailoring protocols, have further increased survival chances in low- and intermediate-risk RMS. To date, the most relevant advances in RMS treatment have been (i) the omission of radiotherapy and alkylating agents in low-risk patients, (ii) the inclusion of upfront irinotecan (in the US) for intermediate-risk patients to reduce alkylating agent dosage and its inclusion (in Europe) in relapsed/refractory cases with temozolomide; and (iii) the use of maintenance therapy to prevent tumour relapse. Nevertheless, high-risk patients still fare poorly, and survival rates remain very low (<30%). The intensification of chemotherapeutic regimens has not translated into better responses to treatment. Knowledge of drug resistance mechanisms upregulated in response to chemotherapy could potentially be harnessed to identify new drug combinations that evade or circumvent such resistance, with clinical benefits to high-risk RMS patients. Improved knowledge of DMEs, especially in young patients, where the enzyme expression is likely to fluctuate more during growth and development, could lead to better drug combination choices beyond standards such as VAC and IVA. For example, the detoxifying role of ALDH isozymes in alkylating agent resistance and CYPs in the activation or detoxification of chemotherapeutics need to be further considered and investigated. In the short-to-medium term, we suggest that a focus should be on replacing an agent in existing drug combinations that is not affected by DMEs to combat enzyme-related resistance to widen the therapeutic index and lead to better treatment outcomes. Given the increasing knowledge that exists about RMS disease, the longer-term focus should be shifted towards the development of immunoconjugates, and other targeted therapies, as discussed elsewhere in this themed issue.

## Abbreviations

ALDH	Aldehyde Dehydrogenase
ARMS	Alveolar rhabdomyosarcoma
COG	Children’s Oncology Group
CSC	Cancer stem cells
CYP	Cytochrome P450
CWS	Cooperative Weichteilsarkomen Studie
DME	Drug-metabolising enzymes
EFS	Event-free Survival
EpSSG	European Paediatric Soft Tissue Sarcoma Study Group
ERMS	Embryonal rhabdomyosarcoma
FNRMS	Fusion-positive rhabdomyosarcoma
FPRMS	Fusion-negative rhabdomyosarcoma
IRSG	Intergroup Rhabdomyosarcoma Study Group
IVA	Ifosfamide + vincristine + actinomycin D
PRMS	Pleomorphic rhabdomyosarcoma
RMS	Rhabdomyosarcoma
SIOP-MMT	International Society of Paediatric Oncology–Malignant Mesenchymal Tumour Committee
STS	Soft tissue sarcoma
STSC	Soft Tissue Sarcoma Committee
TME	Tumour microenvironment
VAC	Vincristine + actinomycin D + cyclophosphamide
